# A new platform for ultra-high dose rate radiobiological research using the BELLA PW laser proton beamline

**DOI:** 10.1038/s41598-022-05181-3

**Published:** 2022-01-27

**Authors:** Jianhui Bin, Lieselotte Obst-Huebl, Jian-Hua Mao, Kei Nakamura, Laura D. Geulig, Hang Chang, Qing Ji, Li He, Jared De Chant, Zachary Kober, Anthony J. Gonsalves, Stepan Bulanov, Susan E. Celniker, Carl B. Schroeder, Cameron G. R. Geddes, Eric Esarey, Blake A. Simmons, Thomas Schenkel, Eleanor A. Blakely, Sven Steinke, Antoine M. Snijders

**Affiliations:** 1 Accelerator Technology and Applied Physics Division, Lawrence Berkeley National Laboratory, Berkeley, CA 94720 USA; 2 State Key Laboratory of High Field Laser Physics and CAS Center for Excellence in Ultra-Intense Laser Science, Shanghai Institute of Optics and Fine Mechanics, Chinese Academy of Sciences, Shanghai, 201800 China; 3 Biological Systems and Engineering Division, Lawrence Berkeley National Laboratory, Berkeley, CA 94720 USA; 4 Present Address: LMU Munich, Geschwister-Scholl-Platz 1, 80539 Munich, Germany; 5 Present Address: Michigan State University, East Lansing, MI 48824 USA; 6 Present Address: Marvel Fusion GmbH, Blumenstrasse 28, 80331 Munich, Germany

**Keywords:** Plasma-based accelerators, Laser-produced plasmas

## Abstract

Radiotherapy is the current standard of care for more than 50% of all cancer patients. Improvements in radiotherapy (RT) technology have increased tumor targeting and normal tissue sparing. Radiations at ultra-high dose rates required for FLASH-RT effects have sparked interest in potentially providing additional differential therapeutic benefits. We present a new experimental platform that is the first one to deliver petawatt laser-driven proton pulses of 2 MeV energy at 0.2 Hz repetition rate by means of a compact, tunable active plasma lens beamline to biological samples. Cell monolayers grown over a 10 mm diameter field were exposed to clinically relevant proton doses ranging from 7 to 35 Gy at ultra-high instantaneous dose rates of 10^7^ Gy/s. Dose-dependent cell survival measurements of human normal and tumor cells exposed to LD protons showed significantly higher cell survival of normal-cells compared to tumor-cells for total doses of 7 Gy and higher, which was not observed to the same extent for X-ray reference irradiations at clinical dose rates. These findings provide preliminary evidence that compact LD proton sources enable a new and promising platform for investigating the physical, chemical and biological mechanisms underlying the FLASH effect.

## Introduction

More than half of all cancer patients receive radiotherapy as the current standard of care^1,2^. Improvements in radiotherapy technology over several decades have resulted in increased precision targeting, which has enabled higher doses to be delivered to the tumor while at the same time minimizing dose delivered to surrounding normal tissues. These advances have allowed the field of radiotherapy to advance towards curative treatment^3^. The development of particle beams for tumor treatment played a critical role in this advancement. High-energy ion therapy is unique in that unlike conventional radiation modalities, which show high entry doses and diminished dose at depth, ion doses are primarily deposited in an inverse way in a narrow range at depth called the Bragg peak following a low entrance dose, thus sparing surrounding normal tissue in front of and behind the tumor volume. The main limitations for the use of ion therapy severely hinder world-wide patient access. Limitations include size and cost of building and maintaining the required accelerator facilities and treatment planning, which is more technically demanding for ion therapy compared to conventional photon-based therapy. These issues are limiting the assessment of the radiobiological potential of proton and heavier ion beams for clinical radiotherapy. Novel technologies that reduce both the accelerator footprint and operating costs are currently being developed. Laser-driven (LD) ion sources are receiving increasing attention due to their potential of providing high-quality proton beams for radiation oncology on a relatively small footprint compared to conventional particle therapy facilities^4–7^. However, our current knowledge about the biological effectiveness of LD ion beams is relatively limited^8–13^.

Recently, published evidence for beneficial differential effects on tumors versus normal tissues using the delivery of single, high radiation doses of > 10 Gy at extremely high mean dose rates (MDR, > 40 Gy/s with doses > 10 Gy delivered in < 100 ms), has been re-visited after decades of previous anecdotal reports, and termed the “FLASH radiotherapy effect”^14^. The FLASH radiobiological or radiotherapy effect requires identification of specific radiation delivery capabilities, including the mean and instantaneous dose rate, dose per pulse, pulse repetition rate, pulse duration, field size and volume, fractionation schedule, and the ambient oxygen tension at the biological target^15^. A recent review summarizes the beam parameters for typical electron beam and proton mini-beam FLASH effects^16^. The FLASH effect has now been successfully demonstrated experimentally in vivo in mice^17,18^, pig and cats^19^, and in a first in-human application to treat superficial tumors^20^. However, radiobiological research into the mechanism of the FLASH effect at high proton MDR has been limited by the restricted access to proton facilities to take advantage of the inverse depth dose profile for in vitro and in vivo experiments^21,22^. As such, there is much speculation regarding the underlying molecular and cellular mechanisms at play for irradiations with FLASH proton doses^16,23,24^ and current research has revealed mixed information about whether the FLASH effect was induced or not^25,26^. Importantly, there is a lack of in vitro studies with normal cells at FLASH proton dose rates at clinical doses^22^.

Due to their generation mechanism, LD ion beams feature ultra-high instantaneous dose rates (IDR) due to ultra-short picosecond pulse lengths generated at the source^27^. At PW laser pulse repetition rates of at most 1–10 Hz, a moderate MDR is achieved. During beam transport to the irradiation site the pulse duration spreads in time leading to nanosecond to few 10 ns long pulses resulting in > 10^7^ Gy/s IDR available for irradiation, several orders of magnitude higher than typically delivered with conventional accelerator technology. These characteristics make LD ion sources highly suitable for a compact experimental platform to study the in vitro and in vivo radiobiological effects of ultra-high IDR protons. Few in vitro studies have been conducted investigating these effects with LD protons, of which all were performed at fully aerobic ambient ~ 20% oxygen levels^28^. So far, beamlines for LD protons for radiobiological studies^8–12,29^ were operated at low repetition rates^9,10,29^ or delivered the dose in a relatively small lateral field with limited tunability^11^.

Here we present a new multidisciplinary research platform to investigate the radiobiological effects of ~ 30 ns long ultra-high IDR (> 10^7^ Gy/s) proton bunches at ~ 2 MeV, that were accelerated at 0.2 Hz using the Berkeley Lab Laser Accelerator (BELLA) petawatt (PW) laser and delivered to a 10 mm diameter irradiation field by means of an active plasma lens. To our knowledge, these proton beam parameters that are particularly suited for radiobiological in vitro studies at ultra-high proton IDR, are unique both among conventional and laser-driven proton sources. The BELLA PW laser at LBNL is a pioneering system in particle acceleration based on high-intensity lasers^30–32^. We describe our new compact and tunable active plasma lens beam line that is used to transport ions from the laser-target interaction zone to a custom-built cell culture chamber to irradiate normal and tumor human prostate cells in vitro. Pilot experiments applying clinically relevant doses ranging from 7 to 35 Gy at ultra-high IDR showed that LD proton irradiation at 7 Gy and higher efficiently killed prostate tumor cells irradiated in vitro, whereas a significant fraction of irradiated normal human prostate cells survived. Reference irradiations with 300 kVp X-rays at clinical dose rates were conducted and did not show these differential effects to the same extent. RNA-sequencing was used to investigate the transcript profiles of cells at the time of irradiation and identified nine genes associated with cellular and oxidative stress upregulated in prostate tumor cells, which were downregulated in normal prostate epithelial cells.

## Results

### High repetition rate, ultra-high instantaneous dose rate, laser-driven proton beamline

LD proton sources are highly attractive for radiobiological studies with ultra-high IDR, as the accelerated proton bunches feature extremely high particle numbers and very short bunch lengths. However, delivering these proton bunches to a large lateral sample area in a reliable and stable fashion has remained a great challenge.

**Figure 1:**
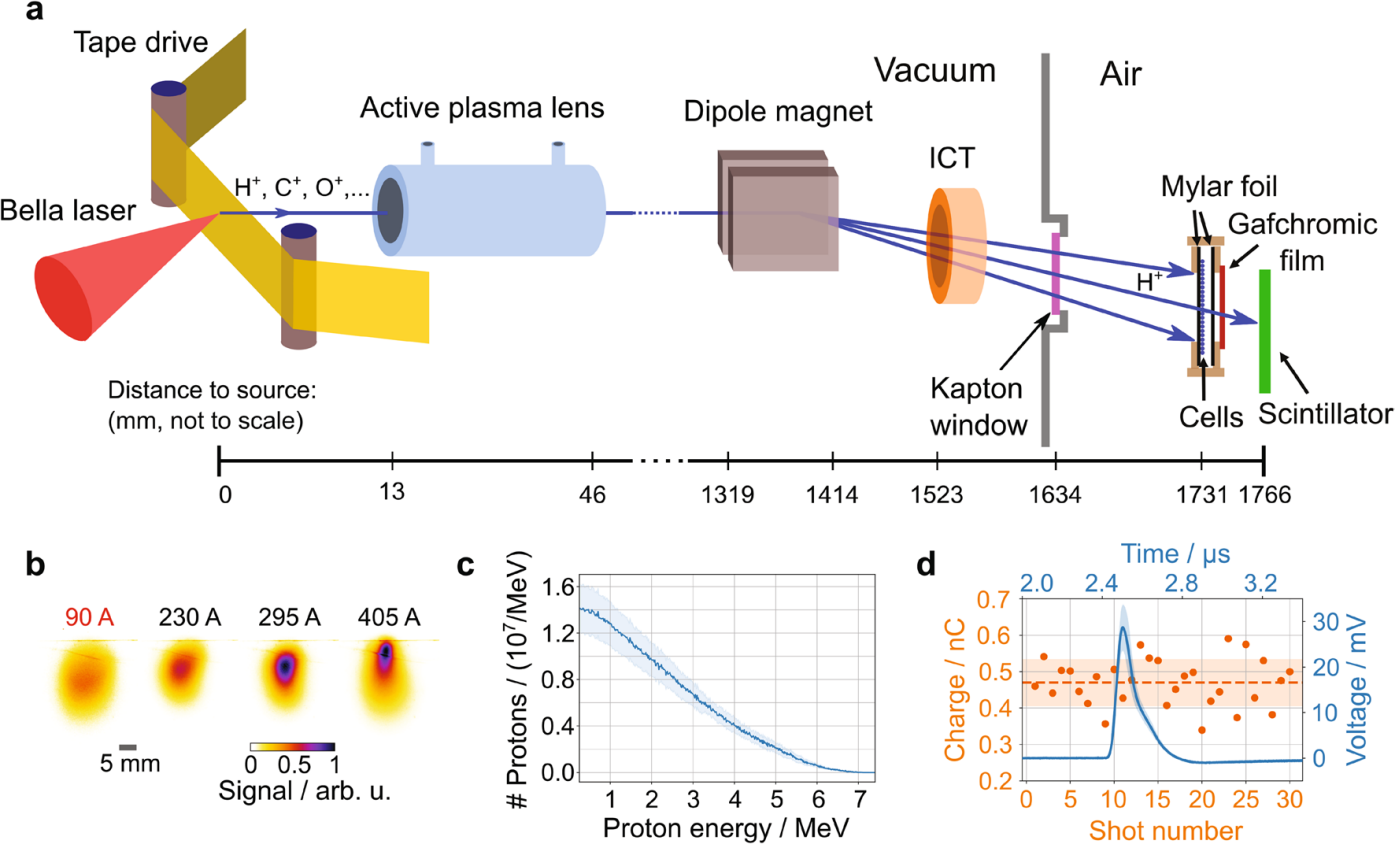
The high repetition rate, ultra-high instantaneous dose rate, laser-driven proton beamline. (**a**) Schematic depiction of the laser-driven proton beamline at the BELLA PW laser. (**b**) 2D spatial distribution of the proton beam measured with the scintillator screen at the location of the cell sample, with four different discharge currents applied to the active plasma lens. All cell irradiations were carried out at 90 A. (**c**) Proton spectra applied to the cell samples derived from beam transport simulations. The mean proton energy is 2.4 MeV. Shaded areas represent the standard deviation from shot-to-shot fluctuations. (**d**) Online ion beam charge measurement (orange markers) by the integrating current transformer (ICT) of 30 consecutive shots at 0.2 Hz. The dashed orange line represents the average charge and the blue curve the averaged ICT voltage signal. Shaded regions correspond to the standard deviation.

Here, we demonstrate a fully plasma-based tunable LD proton beamline (Fig. 1a), that is the first one to rely on an active plasma lens for beam transport and that allowed us to deliver ~ 30-ns proton bunches to radiobiological cell samples with a homogeneous dose distribution over a > 10 mm diameter spot size and an average dose of 1.0 Gy per shot, resulting in an IDR of 3 × 10^7^ Gy/s. Implementation of a custom-designed tape drive target system at the BELLA PW laser^32^ allowed for high repetition rate proton acceleration up to 1 Hz^33^. The BELLA PW laser was used to deliver pulses with 35 J pulse energy and 45 fs pulse length to the tape drive target located in the laser focus of 52 µm diameter at an incidence angle of 45°. Protons were accelerated via target normal sheath acceleration (TNSA)^27^ and featured a broad energy distribution with a high energy cut-off beyond 7 MeV as reported in a previous work^33^. A compact Argon-filled active plasma lens (APL)^34^ with lateral outer dimensions of 50 mm × 40 mm, a length of 33 mm and a channel diameter of 1 mm was placed at 13 mm behind the tape drive target and was used to collect and transport the protons downstream. Through a capillary discharge, the APL can generate a strong, tunable and radially symmetric magnetic field gradient up to 600 T/m to focus charged particles at high repetition rates. The applied discharge current of the APL was optimized to 90 A to reduce the divergence of the captured proton beam fraction and to provide a uniform 10 mm diameter beam spot at the location of the radiobiological cell samples, at 1766 mm from the proton source. Different proton beam spot diameters and thus proton intensities at the irradiation site can be generated on-demand by tuning the APL discharge current (Fig. 1b). Radiochromic film (RCF) stack measurements of the particle number before and after the APL showed a transport efficiency of ~ 0.2% for protons of > 1.5 MeV in-vacuum energies. In addition, a 264 mT dipole magnet with 138 mm effective length was introduced in the proton beam path after the APL to deflect the protons downward from the laser plane and avoid direct irradiation of cell samples by electrons, X- or gamma-rays. Due to this irradiation geometry, the upper edge of the cell cup holder casts a shadow on the cell cup resulting in a crescent-shaped area of 4 mm^2^ of the cell samples that remained un-irradiated. Note that, although 1 Hz operation is possible for the laser, the radiobiological experiments presented in this report were carried out at a reduced repetition rate of 0.2 Hz due to current tape-drive target replenishment limitations.

The proton beam exited the vacuum chamber system through a 25 µm thick Kapton window, which simultaneously filtered any remaining heavy ions originating from the target and passing through the APL. The sealed cell samples were located in air, 97 mm after the Kapton window. Based on the APL properties and in-vacuum spectrum measurements by RCF stacks after the APL, the two-dimensional (2D) on-sample proton spectrum was simulated, showing that both charge density and spectral distribution were uniform across the cell sample. Therefore, no significant lateral dose variation resulted from the use of the dipole magnet (Supplementary Fig. 1). Shown in Fig. 1c is a spatially integrated 1D proton spectrum for the 10 mm diameter irradiation field. The effects from geometries and energy loss summarized in the Supplementary Table 1 were taken into account. Time of flight analysis indicated that the on-cell proton bunch length was ~ 30 ns.

Absolute doses for each irradiated cell sample were measured in situ with a single radiochromic film (RCF) attached to the back of each cell sample cup. Online diagnostics served for efficient tuning and monitoring of the proton beam performance. An integrating current transformer (ICT) was placed behind the dipole magnet and recorded the total charge of the ion bunches during the cell sample irradiation. Long-term stable beam performance was established over the course of the cell irradiation campaign, resulting in an average charge of (0.41 ± 0.06) nC per shot and a shot-to-shot variation of (14.4 ± 4.9)% (standard deviation). Figure 1d displays beam charge measurements over 30 consecutive shots. Finally, the 2D spatial distributions of the proton beams after their propagation through the whole system were recorded by means of a scintillator screen placed at the end of the beamline. This scintillator was also used to initially tune the beam spot distribution at the location of the cell samples as displayed in Fig. 1b. In summary, we developed a compact, high repetition rate, tunable LD proton beam line with in situ absolute dosimetry for radiobiological experiments at ultra-high proton IDR.

### Cell culture assembly and stage design for proton irradiation of monolayer cell cultures

**Figure 2:**
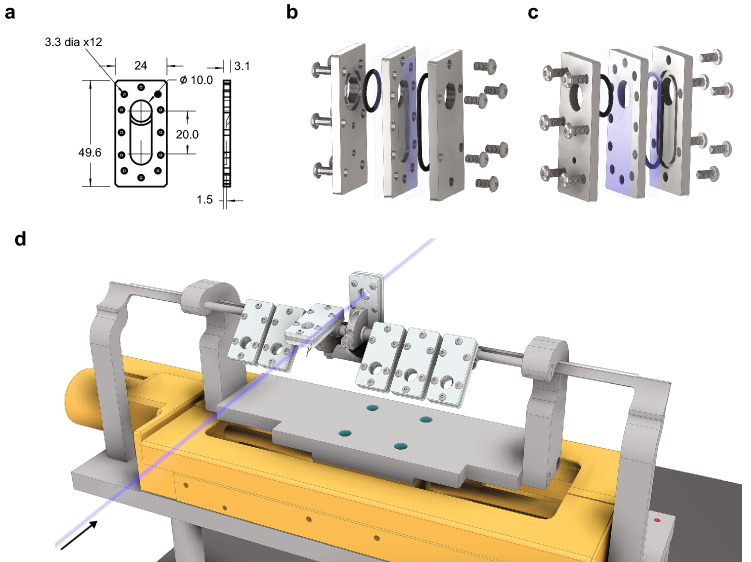
Cell irradiation assembly. (**a**) Stainless steel cartridge containing a 10 mm circular window. Dimensions are provided in mm. (**b**,**c**) Exploded view drawings of cell culture assembly viewed from right (**b**) and left (**c**). Mylar film is depicted in translucent blue. The entire assembly is closed using 12 screws. (**d**) Linear stage holding up to seven cell culture assemblies at − 135° to ensure cell culture media covering the cells. The proton beam is shown using a translucent blue line and the black arrow indicates the direction of propagation of the beam. Each assembly rides up a ramp, lifting the assembly to the upright position allowing cell culture media to drain into the cavity clearing the path for the proton beam to pass through the window.

We designed and built a low-cost and re-usable cell culture holder providing a circular cell irradiation field with a diameter of 10 mm. Each holder consists of a 49.6 × 24 × 3.1 mm stainless steel cartridge with a 10 mm circular open window and a 10 mm wide groove down the center of the window (Fig. 2a; Supplementary Video 1). To provide a surface for the growth of the cell monolayers, mylar film (3.6 µm in thickness) was mechanically stretched over each side of the cartridge and held in place by an aluminum top and bottom seated with rubber gaskets to prevent cell culture media leakage. The entire assembly is held together using 12 screws providing a leak-proof cell culture holder (Fig. 2b,c; Supplementary Video 2). The maximum volume of cell culture media in each well is 270 µl, and when the holder is lifted in the upright position for proton irradiation, the media fills the adjacent cavity created underneath the window, thus clearing the path for the proton beam to enter and exit the chamber through the mylar film to which the cells are attached keeping them sterile while allowing for beam characterization downstream of the cell targets. A linear motorized stage was built to hold up to eight assembled cell culture assemblies at an angle of incidence of − 135° to ensure cell culture media covered the cells (Fig. 2d; Supplementary Video 3). Immediately before irradiation, each individual holder was remotely moved in position and mechanically lifted on a ramp to 0° to allow the cell culture media to fill the cavity leaving the cells with only a thin film of media, and allowing for the proton beam to pass through the entrance mylar window, expose the cells, and pass through the exit mylar window onto RCF film for dose measurements. After exposure, cell culture assemblies were remotely laid down to restore full media coverage, removed from the stage and were held still sealed off from ambient air at 37 °C for 24 h. We developed a cost-effective and re-usable cell culture assembly for proton irradiation of monolayer cell cultures using a mylar membrane cell culture substrate.

### Dosimetry results for cell irradiation with laser-driven protons and reference X-rays

**Figure 3:**
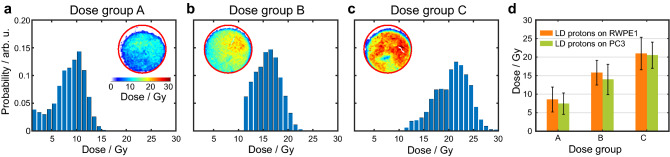
Dosimetry results of laser-driven proton beams and reference X-rays. (**a–c**) Examples of the dose distribution across the cell samples for different dose groups in the form of histograms and 2D color plots (insets, where the 10 mm cell cup diameter is indicated by red circles) of laser-driven (LD) protons. For the proton dose histograms, the non-irradiated crescent-shaped shadow area, visible in the upper part of the insets, was excluded (refer to the Methods section for details). (**d**) Summary of average doses from the dose groups displayed in (**a**–**c**).

Human normal and tumor prostate cells were exposed to LD protons or to reference X-rays and the results were compared. Dose values for all irradiated cell samples are listed in Supplementary Table 2. For LD protons, the dose distribution was measured in situ by calibrated RCF (HD-v2, Gafchromic) attached to the back of the cell holders. Figure 3a–c show typical dose histograms and the corresponding 2D dose distributions, displayed as insets and Fig. 3d shows total dose from each group. A correction factor that was derived from SRIM simulations^35^ of the setup and that took into account the proton spectrum both at the location of the cells (Fig. 1c) and the RCF, was applied to the measured dose to account for differences in absorber material at the location of the cells as compared to the location of the RCF layer. An average dose of 1.0 Gy per shot was delivered to the cell sample, with a lateral variation of (19.3 ± 5.4)% across the irradiated portion of the cell samples and a variation of (14.3 ± 8.1)% from sample to sample in the same dose group. This yields an IDR of (3.0 ± 0.5) × 10^7^ Gy/s based on the estimated proton bunch length of ~ 30 ns. Four different dose groups, ranging from 7 to 35 Gy, were chosen for the cell irradiation experiment, with the doses delivered by varying the total number of proton bunches (between 10 to 30 shots) entering the cell samples at 0.2 Hz repetition rate to achieve the desired total dose. Considering total irradiation times at this repetition rate, moderate MDR of (0.20 ± 0.03) Gy/s were applied.

X-ray dosimetry was completed with similar techniques. A 300 kVp X-ray tube was continuously operated at 10 mA resulting in an instantaneous dose rate (equivalent to mean dose rate due to continuous wave operation) of 0.022 Gy/s, and a NIST-calibrated ion chamber was used to determine the target exposure time of each X-ray dose for a sample location at 500 mm from the source. To conduct the reference dosimetry, a single RCF layer was placed inside the cell sample holder at the cell sample location and was irradiated separately from the cell samples with the same X-ray tube settings. Total X-ray doses ranging from 0.5 to 10.5 Gy and a dose variation across the irradiation field below 1% were confirmed by the RCF measurement. This dosimetry protocol enabled reliable in situ dose measurements of the proton and X-ray doses delivered to the cell samples for each cell line.

### Increased survival of normal human prostate cells irradiated with laser-driven protons compared to reference X-rays

In a pilot study conducted at our new LD proton beamline, we compared the radiobiological effectiveness of LD ultra-high IDR protons applied to normal and tumor cells. Reference irradiations were conducted with X-rays at clinical dose rates. We seeded 10^5^ PC3 prostate tumor cells and 10^5^ RWPE1 normal prostate epithelial cells into the cell culture assemblies, without sealing the chambers using the top aluminum cartridges. Cells were incubated at 37 °C for 24 h to allow for cell attachment to the mylar, after which the media was replaced with 270 µl fresh culture media to feed the cultures and to remove any non-attached cells. The chambers were then sealed with the top aluminum cartridge and incubated at 37 °C for another 24 h to allow cellular consumption of the oxygen levels and establishment of an equilibrated microenvironment, a technique that has been previously used to demonstrate ultra-high dose rate effects^36^. Confluent cultures were exposed to LD protons (RWPE1: at doses of 8.5, 15.8, 21.0, 37.1 Gy; PC3: at doses of 7.4, 14.0, 20.5, 33.9 Gy) with six biological replicates per dose, or X-rays (at doses of 0.5, 1.0, 2.1, 5.3, 8.3, or 10.5 Gy) with two or three biological replicates per dose in addition to sham controls. To allow a uniform time of processing of all samples after exposure, twenty-four hours after radiation exposure chambers were opened to ambient air, and cells were re-plated in triplicate into 100 mm dishes to determine the surviving fraction. Two weeks after re-plating, surviving colonies were stained using crystal violet and quantified using an automated colony counter system.

**Figure 4:**
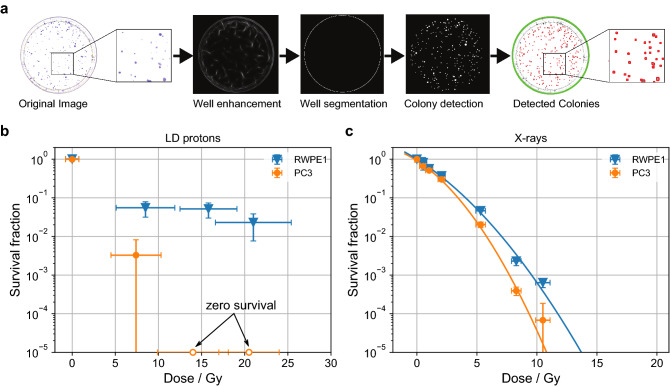
Cell survival fraction based on clonogenic survival. (**a**) Quantitative pipeline for automatic colony quantification. (**b**,**c**) Cell survival fraction based on clonogenic survival of human prostate cancer cells (PC3, orange markers) and normal adult human prostate cells (RWPE1, blue markers) after irradiation with laser-driven protons (**b**) and low dose rate X-rays (**c**). Survival fractions represent the ratio of the plating efficacy of the irradiated cells to unirradiated cells. Error bars of the survival fractions are the standard deviation across two independent experiments, where cell samples were irradiated in triplicate per dose group. Dose error bars contain average non-uniformity in terms of the standard deviation of the lateral dose distribution for each sample, the sample-to-sample variation (standard deviation) within one dose group and the uncertainty arising from the film calibration. Open circles (PC3 only) indicate a survival fraction of zero. An orthogonal distance regression fit with model function y = c·exp(− a·x − b·x^2^) was applied to the X-ray survival fractions. The resulting fit parameters for RWPE1 are c = (0.998 ± 0.029), a = (0.452 ± 0.060), b = (0.028 ± 0.008) and for PC3 are c = (0.917 ± 0.069), a = (0.456 ± 0.081), b = (0.056 ± 0.011).

The number of surviving colonies per cell culture dish provides a quantitative characterization of the dose-dependent survival once normalized to the plating efficiency of the sham-treated controls and allows for statistical evaluation of the impact of LD protons and reference X-rays on radiobiological effectiveness in vitro. We developed a high-throughput quantitative pipeline for automatic colony quantification with high efficiency and effectiveness. This pipeline (Fig. 4a) operates on digital scanned cell culture plate images of surviving colonies on each petri dish plated, followed by two consecutive steps: (1) well detection via ellipse fitting^37^ based on regularized well signal through iterative tangential voting^38^; and (2) colony detection based on dark elliptic features^39^.

For each triplicate cell plating, the plating efficiency was calculated based on the number of cells that were seeded. Plating efficiencies were then normalized to the plating efficiencies observed in sham irradiated controls to calculate the surviving fraction for each of three plates across three (X-ray) or two (proton) independent experiments. For LD proton irradiations, a lethal dose of proton exposure (> 30 Gy) was used to estimate the background surviving fraction due to the irradiation geometry of the proton irradiation setup, which we subsequently subtracted from the surviving fraction of samples irradiated with all proton doses (Supplementary Table 2). The individual samples were then grouped according to applied dose. Survival fractions for LD proton irradiations are displayed in Fig. 4b, excluding the samples that were irradiated with a lethal dose of > 30 Gy. Normal cells (RWPE1) consistently displayed significantly higher survival than tumor cells (PC3) for all doses applied. At 7 Gy, normal cell survival was more than one order of magnitude higher than tumor cell survival. At higher doses, normal cell survival was slightly reduced while no surviving tumor cell colonies were observed. X-ray reference irradiations (Fig. 4c) at clinical dose rates showed a mildly differential survival of normal versus tumor cells that was far less pronounced than observed with LD protons.

In conclusion, our first experiments at the newly developed LD proton beamline at the BELLA PW laser showed increased survival of normal prostate cells compared to prostate tumor cells when irradiated with LD protons at ultra-high IDR. Reference X-ray irradiations at clinical dose rates did not show a similarly pronounced differential survival.

### RNA-sequencing of normal and tumor prostate cells in ambient air and sealed conditions

**Figure 5:**
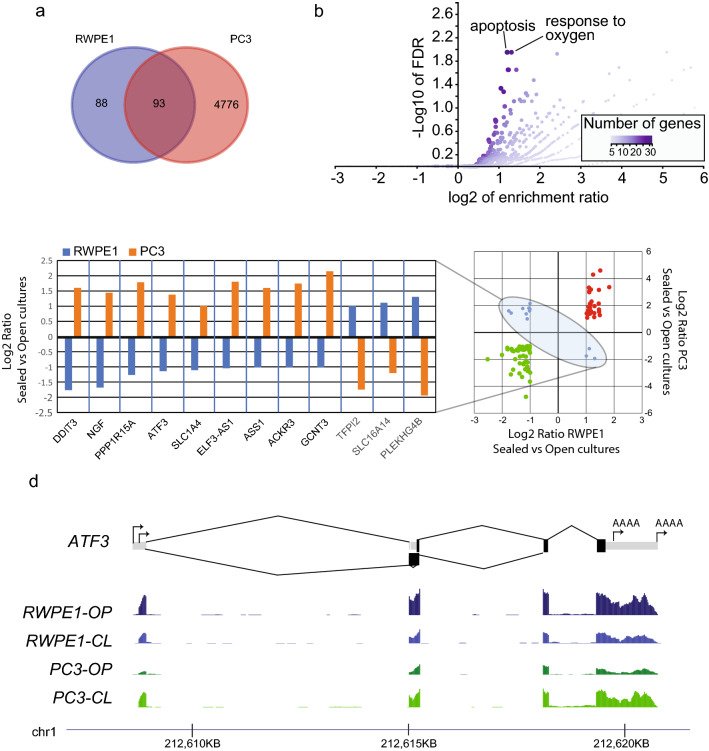
RNA-sequencing analysis of RWPE1 and PC3 cells cultured under sealed and unsealed conditions. (**a**) Number of differentially expressed genes in sealed versus unsealed culture conditions for RWPE1 and PC3 cells. (**b**) Gene ontology enrichment analysis of differentially expressed genes in RWPE1 cells cultured under sealed versus unsealed conditions. (**c**) Gene expression levels of genes differentially expressed in RWPE1 and PC3 cells cultured under sealed versus unsealed conditions. (**d**) ATF3 gene structure and transcriptional profiles for RWPE1 and PC3 cells cultured under sealed (CL) and unsealed (OP) conditions.

In order to better understand the transcriptional profile of RWPE1 and PC3 cells in the sealed cell culture assemblies at the time of irradiation, we performed an RNA-sequencing experiment comparing gene expression to cells cultured in cell culture assemblies exposed to ambient air (non-sealed). We seeded cells in triplicate in our cell culture assemblies and 24 h later cells were either sealed (n = 3 for each cell line) or not sealed (n = 3 for each cell line) from ambient air. RNA was harvested from all wells 24 h later (corresponding to the time of irradiation) for RNA sequencing. We observed a large difference in the number of genes that were differentially expressed between sealed and non-sealed samples. In RWPE1, we observed 181 genes, whereas for PC3, close to 5000 genes were differentially expressed in sealed versus unsealed growth conditions (Fig. 5a). Interestingly, RWPE1 exhibited significant downregulation of genes enriched in oxidative stress functions (Fig. 5b). PC3, on the other hand, showed the opposite response and showed upregulation of nine stress response-related genes that were downregulated in RWPE1 (Fig. 5c), including the oxidative stress responsive transcription factor ATF3 (Fig. 5d). These results suggest that the transcriptional state of RWPE1 at the time of irradiation was characterized by low-oxidative stress, whereas PC3 cells experienced increased stress. Tumor cells typically demonstrate a higher prevalence of stress markers compared to normal tissues^40^.

## Discussion

Size, cost and technical complexity of conventional accelerators are among the factors that limit worldwide access to particle beam radiotherapy^4,6^. Particle acceleration based on high-intensity lasers is a promising approach to develop more compact and cost-effective ion therapy facilities that can be integrated into existing clinical radiotherapy settings^41^. Already at current performance levels, compact LD proton sources could answer the need for, otherwise limited, detailed radiobiological studies investigating the effectiveness and underlying mechanisms of the FLASH effect for proton radiotherapy.

Few platforms that provide laser-driven protons for radiobiological studies have been developed^8–12,29,42^. However, these systems operate at lower repetition rates, are less tunable, or deliver a smaller lateral proton distribution. In this work, by combining the tape drive target with an active plasma lens (APL), we demonstrated a compact, tunable beamline to deliver high repetition rate LD proton pulses at 0.2 Hz to an irradiation field of 10 mm diameter for radiobiological experiments in an ultra-high IDR regime. Stable beam delivery of ~ 2 MeV protons at 1.0 Gy per shot with a dose variation of (14.3 ± 8.1)% between samples of the same dose group and a lateral dose variation of (19.3 ± 5.4)% was established. To our knowledge, such beam parameters have not yet been demonstrated with a LD proton source. We used this compact beamline for the irradiation of radiobiological cell samples with ultra-high IDR proton pulses of (3.0 ± 0.5) × 10^7^ Gy/s to study the differential sparing of healthy tissue that is indicative of the FLASH effect.

We observed a significant difference in radiosensitivity of normal prostate cells versus prostate tumor cells irradiated with ultra-high IDR pulses of LD protons. Reference irradiations with X-rays at clinical dose rates did not show a similarly differential radiosensitivity. More specifically, more than one order of magnitude higher normal than tumor cell survival was observed for irradiations with 7 Gy LD protons. At higher doses, appreciable normal cell survival was observed while no surviving tumor cell colonies were found. This differential sparing of normal cells under ultra-high IDR irradiation could minimize normal tissue toxicity when translated into the clinic. Prior studies did not observe ultra-high IDR induced differential effects using LD protons in cell killing, generating double-strand breaks and other radiation-induced effects^8–12,25,42–45^. However, those studies either focused exclusively on tumor cells or applied total doses no higher than 5 Gy, while we observe the differential sparing of normal cells for doses of 7 Gy and higher, which is in agreement with previous studies observing the FLASH effect^16^. Our experimental conditions were further different from previous in vitro studies, in that we sealed our cell culture assemblies from air 24 h prior to irradiation to attain physiological equilibrium, and re-seeded cells for clonogenic survival analysis 24 h after irradiation exposure with no evidence of cell death in unirradiated controls due to the 48 h of being sealed off from ambient air. These conditions could indicate that the potential depletion of oxygen in our experimental conditions affords protection from ultra-high IDR proton pulses in normal cells, but not tumor cells, perhaps due to differences in metabolic pathways involved in the response of normal and tumor cells to the stress of low oxygen levels. RNA-sequencing of RWPE1 and PC3 cells in the sealed cell culture assemblies at the time of irradiation, compared to gene expression to cells cultured in cell culture assemblies exposed to ambient air (non-sealed) identified nine genes that were upregulated in PC3 and downregulated in RWPE1. Interestingly, the oxidative stress-responsive transcription factor ATF3 was downregulated in RWPE1 and upregulated in PC3 in the sealed chambers. ATF3 is known to be responsive to reactive oxygen species^46^. NGF was similarly differentially expressed and is known to elicit a protective effect against oxidative stress^47^. Taken together these data suggest a possible role for oxidative stress in the observed difference in cell survival of normal and tumor cells after ultra-high IDR LD proton irradiation.

The effects of oxygen on radiation sensitivity in cells has been widely investigated and increased radiosensitivity is observed as the oxygen concentration is increased from anoxia to the 20% oxygen concentration in air^48^. However, the oxygen effect has most often been seen as a means to increase tumor cell killing through re-oxygenation, since most tumors are not well oxygenated when compared to normal, well vascularized, tissues. Yet, many normal cells in the body are routinely at much lower physiological levels of oxygen near 1%^49^. Recently, however, the role of oxygen in radiation sensitivity has been suggested as a possible mechanism for the observed reduction in normal tissue injury after irradiation exposure at ultra-high dose rates^14,50^. These results are in agreement with studies conducted in the late 1960s, which showed increased survival of mammalian cells irradiated at FLASH dose rates^36,51^. Berry irradiated human and hamster cells with a single 7 ns X-ray pulse compared to conventional dose rate exposures using ^60^Co gamma rays^36^. Interestingly, in this study, cell culture flasks were sealed 24 h prior to radiation exposure and the media was replaced 24 h after irradiation, similar to our 48 h sealed off experimental conditions. A more recent study compared radiosensitivity of the prostate cancer cell line DU145 to 10 MeV electrons delivered at FLASH (600 Gy/s; 3 Gy per pulse at 200 Hz) or conventional (0.23 Gy/s) dose rates across different oxygen concentrations^52^. This study showed an oxygen concentration dependent increase in cell survival after FLASH electron exposures of 18 Gy. Interestingly, in our setup, while our instantaneous dose rates are in the order of 10^7^ Gy/s, our mean dose rate is only 0.20 Gy/s considering a pulse separation of 5 s. Using this setup, we observed significant tissue sparing for normal prostate cells, which was not observed for the PC3 prostate tumor cell lines, showing promise in potentially widening the therapeutic window using laser-driven proton beams.

A limitation of this first study lies in the fact that an area containing a few percent of cells was not proton irradiated due to a non-optimal irradiation geometry, which needed to be accounted for in the subsequent analysis of clonogenic survival and the dosimetry. However, the uncertainty arising from subtracting the unirradiated cells from the overall surviving fraction was found negligible compared to the variation of the surviving fraction between sample replicates. Although non-linear bystander effects of unirradiated cells could potentially affect clonogenic survival, we do not expect that this effect can account for the substantially higher survival of normal cells compared to tumor cells observed after LD proton exposure.

Future irradiation studies at the BELLA LD proton beamline will improve irradiation conditions by developing technologies to overcome current limitations of the platform. The lateral dose uniformity will be further improved by optimizing the beamline setup and operation parameters and irradiation of the full cell cup will be ensured. Engineering alterations to the tape drive target will enable pushing the proton pulse repetition rate from 0.2 to 1 Hz to fully match the repetition rate of the BELLA PW laser. Since we seal our chambers from ambient air 24 h prior to radiation exposure, we speculate that cells are exposed to reduced oxygen levels. For future experiments, an oxygen probe will be added to our cell culture holders to assess absolute levels to investigate this. Moreover, molecular studies are planned to further evaluate differential gene expression in normal and tumor cells that might elucidate changes in oxygen metabolic pathways that may contribute to the differential normal cell sparing and tumor cell killing. Reference measurements at clinical dose rates will be extended to include irradiations with proton beams from a conventional (radio frequency driven) accelerator facility, to account for differences in radiobiological effectiveness expected between protons and X-rays. Moreover, our results indicate that ultra-high *instantaneous* proton dose rates could result in differential normal cell sparing, even though the applied *mean* dose rate was far below the previously reported minimum of 40 Gy/s to observe this beneficial effect. Further cell lines with additional biological endpoints will be investigated at different pulse repetition rates, and hence, different mean dose rates, aiming to improve our understanding of the underlying mechanism and optimal conditions for the differential normal cell sparing observed in our study. Finally, in vivo studies to determine the efficacy of LD protons for tumor eradication and normal tissue sparing will be pursued at higher proton energies. This will be enabled by a new experimental installation at the BELLA PW laser that will deliver ~ 30 MeV protons for irradiation studies with in vivo models.

In conclusion, by combining a tape drive target system and an APL for proton beam transport, we established a high repetition rate laser-driven proton beamline, that is capable of delivering ultra-high instantaneous dose rate proton bunches with a quasi-homogeneous dose distribution over a lateral area of 10 mm diameter. Using this beamline, we irradiated in vitro biological cell samples to investigate the radiobiological effectiveness of ultra-high IDR protons. By comparing cell survival fractions of normal versus tumor cell samples and referencing them to X-ray irradiations at clinical dose rates, we find that the differential sparing of healthy tissue whilst inducing substantial tumor cell killing is induced by laser-driven protons at potentially reduced oxygen levels in vitro for doses of 7 Gy and higher. These results, in combination with the low-cost and small-footprint nature of laser-driven proton sources, provide evidence to demonstrate the capabilities of this new platform for elucidating the mechanism and optimal conditions of ultra-high dose rate proton therapy.

## Methods

### Laser system

The experiment was performed using the BELLA PW laser facility at LBNL. The BELLA PW laser was the world’s first 1 Hz repetition rate 1 PW Ti:Sapphire laser system based on double-chirped pulse amplification architecture, where a cross-polarized wave (XPW) contrast enhancement system is installed in between two CPA stages, delivering pulses with a duration down to ~ 35 fs FWHM at 815 nm wavelength. A 13.5 m focal length off-axis parabolic mirror is used to focus the laser pulses with around 35 J energy to a measured spot size of 52 μm FWHM diameter, yielding a peak intensity of 12 × 10^18^ W/cm^2^. For this experiment the laser was operated at 45 fs pulse length, optimized for maximum proton energy. Although 1 Hz operation would be possible, the experiments were performed with a repetition rate of 0.2 Hz.

### Tape-drive target

Kapton tape with a thickness of 13 µm was irradiated with a 45-degree angle of incidence. In our target assembly, the Kapton tape is spooled into a feedback-controlled tape drive system, and continuously moved by two DC-motors, providing a fresh wrinkle-free target surface with a position repeatability < 10 µm. Such a tape drive is capable of operating at a high repetition rate up to 1 Hz.

### Proton transport and diagnostics

The proton pulse was focused using an active plasma lens (APL)^34^, which is a 1-mm-diameter Argon gas-filled capillary with a length of 33 mm. The gas pressure applied inside the capillary was 5 Torr. This compact device provides a radially symmetric focusing force for charged particle beams up to 600 T/m via a capillary discharge current. A 264 mT dipole magnet with 138 mm effective length was used to deflect the protons downward and provide shielding of the cells from secondary radiation. The protons exited through a 25 μm thick Kapton foil window and entered the cell sample holder. The Kapton foil was thick enough to completely stop a potential contribution of heavy ions originating in the laser-target interaction, for example, carbon, oxygen and nitrogen ions.

An integrating current transformer (ICT) was located before the Kapton window, providing online charge measurement of all ions transported by the APL. All ions passing through the ICT contributed to the measured ICT signal, the total bunch charge was then estimated by integrating over the whole signal curve (Fig. 1d). By comparing the ICT measured charge with absolute doses measured with RCF, we could establish their close correlation, rendering the ICT a reliable online beam stability diagnostic. A scintillator, located 35 mm behind the cell sample and imaged to a CCD, allowed for online beam position monitoring. Without the proton beamline (i.e. APL and dipole magnet) in place, a Thomson parabola spectrometer was used to measure energy spectra of protons and other ions that were laser-accelerated from the tape drive target^33^.

In order to optimize and evaluate the proton beam transport, proton energy spectra were measured by using stacks of radiochromic films (RCF, Gafchromic HD-v2) at the following three locations, (1) 30 mm after the tape-drive target, (2) with the APL at 1432 mm from the tape-drive target without the dipole magnet in place, and (3) at the cell sample location at 1731 mm from the tape-drive target with the dipole magnet in place for various APL currents. By comparing the first two spectra, a transport efficiency of about 0.2% was deduced for proton energies above 1.5 MeV (in vacuum). The measurements at the third location showed that by applying a discharge current of 90 A, this geometry produced a uniform > 10 mm diameter beam spot in a plane 1731 mm away from the target as shown in Fig. 1b.

Based on the knowledge of the proton spectra above and magnetic properties of the APL and the dipole, a two-dimensional (2D) on-cell proton spectrum was simulated using the arbitrary order beam physics code COSY INFINITY^53^ and a home-made Matlab script. The APL was modeled as an equivalent quadrupole magnet but modified in order to provide focusing force in both planes. The proton beam at the tape drive was defined with 100 μm (in full-width half-maximum, FWHM) source size and 260 mrad FWHM divergence but limited within 11 mrad due to the acceptance angle of the APL. Instead of modeling proton particles loss dynamically within the APL, the proton spectrum was defined by the aforementioned RCF measurement (location 2), *N*/*dE* = 5.15 × 10^9^ *exp*(− *E*/1.04), where *N* is the number of protons and *E* is the kinetic energy of protons in MeV. This allowed the modeling of the APL as a simple 100% transmission element, while simulating the energy dependent beam convergence accurately. The energy loss and dose calculation were performed with the Matlab script.

The 2D on-cell proton spectrum simulation results are shown in Supplementary Fig. 1a,b. The spectrum includes the effects from geometries and energy loss summarized in the Supplementary Table 1. The simulated proton density was found to be spectrally uniform across the 10 mm diameter spot except proton energies 5.4 MeV and above in the vertical axis, the contribution of which was 0.6%. Furthermore, taking the energy dependent linear energy transfer (LET) range into account (shown in Supplementary Fig. 2), a dose distribution over the 10 mm spot was calculated and shown in Supplementary Fig. 1c, where the standard deviation was found to be 18%. Based on this simulated dose distribution, no significant lateral dose dependence was expected as a result of using of the dipole magnet.

### Dosimetry

The proton dose distribution was individually measured for each sample using calibrated RCFs (Gafchromic HD-v2) placed immediately behind the back layer of the cell container. The films were scanned (EPSON Perfection V600 Photo scanner) in landscape format with all image correction features turned off with a resolution of 1000 dpi in transmission mode and saved as 16-bit grayscale tiff images. Scanning was done several days after irradiation to allow for stabilization of the optical density development post-irradiation. The scanner was calibrated with a NIST-calibrated transparent step wedge to convert the raw data to optical density (OD). The dose applied to the cells was higher than the dose applied to the film due to additional absorbers the protons traversed until they reached the film. This required multiplying the film-measured dose by a correction factor to obtain the dose applied to the cells. SRIM simulations, modeling the energy loss in the additional absorbers (5 μm cell layer, 2995 μm air gap, 3.6 μm mylar window, 3000 μm air gap) between the cell samples and the film, resulted in a correction factor of 0.9. By using TOPAS MC^54^ an independent Monte-Carlo simulation was carried out to study impacts from secondary particles, which SRIM simulations cannot address. The dose introduced by secondary particles was found to be less than 1%, and therefore considered negligible.

The X-ray reference dosimetry was performed separately from the cell irradiation by placing a single RCF (Gafchromic EBT-3) inside the cell cup holder at the location of each cell sample. Films were irradiated in triplicate for each dose at the same X-ray tube settings used for cell irradiations. An unirradiated piece of the same EBT-3 sheet was used as a 0 Gy reference. Films were scanned several days after the irradiation with an Epson Expression 12000XL scanner in transmission mode with a resolution of 600 dpi and landscape format with all image correction features turned off and saved as 16-bit grayscale tiff images.

Both kinds of Gafchromic films used in these studies were calibrated with a 320 kV X-ray Tube (X-RAD320), operated at 300 kV, for doses between 5 and 15 Gy. For HDv-2 film dose vs. netOD = OD-OD_0_ was fitted with a function of the form D = a + b∙netOD with free parameters a and b (a = − 0.388 Gy, b = 434.913 Gy). For EBT-3 the fit function D = a + b∙netOD + c∙netOD^d^ was used (a = 0.012, b = 2.797, c = 3.982, d = 2.784). The calibration data and fit functions for both film types are displayed in Supplementary Fig. 3. In our cell irradiation setup, the protons have lost most of their kinetic energy once they reach the sensitive layer of the RCF, meaning that dose detection involves high linear energy transfer (LET) resulting from Bragg peak stopping of the lowest energy protons reaching the RCF (refer to Supplementary Fig. 2), which leads to reduced sensitivity of RCFs^55^. Therefore, we calculated the energy-dependent correction factor η^56^ based on the LET derived from SRIM simulations, so that D_real_ = D/η. Using the proton spectrum delivered to the RCF determined by the APL beam transport simulation, we applied a weighted average correction factor η = 0.7 to RCF measured doses to account for LET related RCF sensitivity reduction.

### Dose evaluation

Samples that were irradiated with X-rays were exposed to a continuous wave (cw) beam, while samples irradiated with laser-driven (LD) protons were exposed to a sequence of pulses at ultra-high instantaneous dose rates. Table 1 summarizes the RCF dosimetry results for all dose groups applied to the cell samples (dose values for individual samples are listed in Supplementary Table 2). X-ray samples were irradiated as one triplicate per cell line, while LD protons were applied to six or four samples per cell line and dose group. Each scanned film was treated with scanner background subtraction and gray value to OD conversion. The 0-Gy reference background (OD_0_) was subtracted and the images were binned to reduce the influence of noise through dust particles and film irregularities. Due to the irradiation geometry for the proton irradiation study, the upper edge of the cell cup holder cast a shadow on the cell cup resulting in a crescent-shaped area of 4 mm^2^ of the cell sample that remained unirradiated. Geometric propagation of the shadow to the plane of the RCF layer results in a shadow area of 12 mm^2^, visible in the upper part of the lateral dose distribution displayed in Fig. 3. This area was excluded from the dosimetry to appropriately represent the dose distribution applied to the cells. Supplementary Fig. 3 displays a dose histogram with and without the exclusion of the shadow area. The netOD = OD − OD_0_ was converted to dose and the resulting dose distribution was averaged across the irradiated portion of the 10 mm cell cup diameter. Averaging over all irradiated films per dose group results in the values listed in the “mean dose” column in Table 1. The error on the mean dose contains the average non-uniformity in terms of the standard deviation of the lateral dose distribution for each sample, the sample-to-sample variation (standard deviation) within one dose group and the uncertainty arising from the film calibration. The desired dose was obtained by varying the number of proton pulses, or for the X-rays, the exposure time. The dose per laser shot was derived by dividing the mean dose by the number of laser shots, resulting in an average of 1.0 Gy/shot. The instantaneous dose rate of protons was determined by dividing the dose per shot by the proton pulse length of ~ 30 ns, while the mean dose rate values result from dividing by total irradiation time, that is, number of shots divided by repetition rate of 0.2 Hz. X-rays instantaneous and mean dose rates (synonymous due to cw operation) result from dividing mean doses by the total X-ray irradiation time.

**Table 1:** Comparison of dose distribution in each dose group for laser-driven protons and reference X-rays (grey areas are LD proton results and white areas are reference X-ray results).

Radiation type	NR of samples	Mean dose/Gy	Dose error/Gy	Lateral dose variation/%	Dose variation between samples (sd^a^)/%	Fractionation	Total irradiation duration/s	Dose per shot/Gy	Inst. dose rate/Gy/s	Mean dose rate/Gy/s
X-ray RWPE1/PC3	2–3 per cell line	0.5	0.3	< 1%	–	–	24	–	0.022	0.022
X-ray RWPE1/PC3	2–3 per cell line	1.0	0.3	< 1%	–	–	48	–	0.021	0.021
X-ray RWPE1/PC3	3 per cell line	2.1	0.3	< 1%	–	–	93	–	0.022	0.022
X-ray RWPE1/PC3	2–3 per cell line	5.3	0.4	< 1%	–	–	228	–	0.023	0.023
X-ray RWPE1/PC3	2–3 per cell line	8.3	0.4	< 1%	–	–	362	–	0.023	0.023
X-ray RWPE1/PC3	2–3 per cell line	10.5	0.6	< 1%	–	–	450	–	0.023	0.023
LD protons RWPE1	6	8.5	3.4	27	25.9	10	50	0.85	2.6E + 07	0.24
LD protons^A^ PC3	6	7.4	2.9	29	21.1	10	50	0.74	2.3E + 07	0.17
LD protons^B^ RWPE1	6	15.8	3.3	16	11.4	16	80	0.99	3.0E + 07	0.15
LD protons PC3	6	14.0	4.1	16	23.4	16	80	0.87	2.6E + 07	0.20
LD protons RWPE1	6	21.0	4.4	15	13.4	20	100	1.05	3.2E + 07	0.17
LC protons^C^ PC3	6	20.5	3.5	15	5.7	20	100	1.03	3.1E + 07	0.21
LD protons RWPE1	4	37.1	7.1	18	7.5	30	150	1.24	3.8E + 07	0.21
LD protons PC3	4	33.9	6.4	18	6.2	30	150	1.13	3.4E + 07	0.25

A, B, C: examples of histograms and lateral dose distributions are displayed in Fig. 3.

^a^Standard deviation.

### Cell culture

Normal human prostate epithelial cells RWPE1 (CRL-11609) and cancer cells PC3 (CRL-1435) were purchased from ATCC (Manassas, VA). RWPE1 cells were maintained in Keratinocyte Serum Free Medium (K-SFM; ThermoFisher 17005042) supplemented with 0.05 mg/ml bovine pituitary extract and 5 ng/ml human recombinant epidermal growth factor. PC3 cells were maintained in F-12K Medium (ThermoFisher 21127022) supplemented with fetal bovine serum to a final concentration of 10%. All cells were grown at 37 °C with 5% CO_2_ in air. The culture medium was replaced every three days with fresh culture media and cells were sub-cultivated at 70% confluency at a ratio of 1:3.

### Cell preparation for radiation exposures

A 3.6 μm mylar film (Chemplex mylar spectromembrane 3013) was mechanically stretched over the 10 mm irradiation window and screwed in place (Fig. 2b,c). 1.0 × 10^5^ PC3 or RWPE1 cells were seeded in 100 μl culture media on the mylar film over the irradiation window and cultured at 37 °C with 5% CO_2_ in air for three days until 80% confluence. On day four, the culture media was removed and replaced with 280 μl fresh culture media. A second 3.6 μm mylar film was manually stretched over the culture holder, screwed in place (Fig. 2a) and cultured overnight at 37 °C to complete the sample assembly. Twenty-four hours after closing the cell culture holders off from ambient air, cells were irradiated at room temperature and immediately placed back at 37 °C. Twenty-four hours after radiation exposure, the media was removed and cells were detached from the mylar membrane using 100 μl 0.25% trypsin. Trypsin was inactivated with 100 μl soy trypsin inhibitor (ThermoFisher R007100) and cells were collected by centrifugation at 1200 rpm for 3 min. The cell pellet was resuspended in 600 μl culture media and cells were counted using a hemocytometer. Based on the radiation dose, different numbers of cells were seeded in triplicate 100 mm cell culture plastic dishes (Greiner 07-000-386). Culture media was changed weekly and 14 days after seeding, surviving colonies were fixed and stained using crystal violet (0.25% crystal violet in 70% ethanol). All stained dishes were photographed for automated colony detection and quantification.

### Method for well detection

Well detection provides the region of interest (ROI) for colony recognition and characterization, which was achieved with two consecutive steps including well regularization and enhancement followed by well segmentation. The first step is to regularize and enhance the well boundary from the raw photographs using iterative tangential voting^35^, so that the boundary can be accurately detected for ellipse fitting in the next step. The main theme of iterative voting is to infer saliency, which can be in the form of closure, continuity and symmetry. The inference is achieved by specialized kernel design that elucidates a specific feature through iterative refinement. Specifically, in the application to the regularization of well boundary, the boundary signals correspond to the negative curvature maxima at a given scale within the image space, and the details of kernel design and implementation can be found in our previous work^35^. Given the enhanced signal of the well boundary, segmentation is formulated as an ellipse fitting problem with least square fitting strategy^34^. During fitting, the parameters of the well, including location, aspect ratio, rotation, major axis and minor axis are optimized based on the boundary signals, and thereafter, the well region is effectively detected and segmented from the raw microscopic image, which will be utilized as the region of interest for colony detection as described below.

### Colony detection

Given the observation that the colony region is typically darker than the surrounding background in the well, we define the colony regions as the dark elliptic features^39^ within the corresponding well. Let the linear scale-space representation of the original image ${I}_{0}(x,y)$ at scale σ be given by:

1 $$I\left(x,y;\sigma \right)= {I}_{0}\left(x,y\right)\times G\left(x,y;\sigma \right),$$

where $G\left(x,y;\sigma \right)$ is the Gaussian kernel with a standard deviation (SD) of $\sigma$. For simplicity $I\left(x,y;\sigma \right)$ is also denoted as $I\left(x,y\right)$ below. At each point $\left(x,y\right)$, the iso-intensity contour is defined by:

2 $$I\left(x+\Delta x,y+\Delta y\right)= I\left(x,y\right),$$

where $\left(\Delta x,\Delta y\right)$ is the displacement vector. Expanding and truncation of the above equation using Taylor’s series, we have the following estimation:

3 $$\frac{1}{2}\left(\Delta x,\Delta y\right)H\left(x,y\right){\left(\Delta x,\Delta y\right)}^{T}+\left({I}_{x},{I}_{y}\right){\left(\Delta x,\Delta y\right)}^{T}=0,$$

where

$$H\left(x,y\right)=({I}_{xx} {I}_{xy} {I}_{xy} {I}_{yy} )$$

is the Hessian matrix of $I(x,y)$. The entire image domain is divided by Eq. (2) into two parts:

4 $$I\left(x+\Delta x,y+\Delta y\right)> I\left(x,y\right),$$

5 $$\text{and }I\left(x+\Delta x,y+\Delta y\right)< I\left(x,y\right),$$

or locally

6 $$\frac{1}{2}\left(\Delta x,\Delta y\right)H\left(x,y\right){\left(\Delta x,\Delta y\right)}^{T}+\left({I}_{x},{I}_{y}\right){\left(\Delta x,\Delta y\right)}^{T}>0.$$

And

7 $$\frac{1}{2}\left(\Delta x,\Delta y\right)H\left(x,y\right){\left(\Delta x,\Delta y\right)}^{T}+\left({I}_{x},{I}_{y}\right){\left(\Delta x,\Delta y\right)}^{T}<0.$$

If $H\left(x,y\right)$ is positive definite, then the region defined by Eq. (4) is locally convex. Similarly, if $H\left(x,y\right)$ is negative definite, then the region defined by Eq. (5) is locally convex. To determine whether $H\left(x,y\right)>0$ or $H\left(x,y\right)<0$, we analyze the feature in both cases:

1. $H\left(x,y\right)>0$. Then ${I}_{xx}>0$, ${I}_{yy}>0$, and hence ${I}_{xx}+{I}_{yy}>0$, and positive Laplacian means that $(x,y)$ is a “dark point”, i.e., a point that is darker than its neighbors; and,
2. $H\left(x,y\right)<0$. Then ${I}_{xx}<0$, ${I}_{yy}<0$, and hence ${I}_{xx}+{I}_{yy}<0$, and negative Laplacian means that $(x,y)$ is a “brighter point”, i.e., a point that is brighter than its neighbors.

From a computational perspective, we have the following definition: a point is a bright (dark) elliptic feature at scale $\sigma$ if the Hessian matrix of $I(x,y;\sigma )$ is negative (positive) definite at that point. The net result of applying dark elliptic feature detection is a binarized mask corresponding to colony regions and background. However, very small regions may have been created as a result of inherent noise in the image, which are then removed based on size and intensity thresholds.

### Clonogenic survival

After colony counting, we calculated the surviving fraction by dividing the number of colonies by the number of cells seeded. For proton irradiations, the irradiation field did not fill the cell area completely, leaving out a 1.5–2.7% region of cells that were not irradiated. We used a lethal dose of proton exposure (> 30 Gy) to estimate the background surviving fraction, which we subsequently subtracted from the surviving fraction of samples irradiated with all doses. The fraction of surviving cells identified this way matches the size of the geometric shadow region with respect to the total cup size. We then calculated the average surviving fraction for the sham exposures, which was used to normalize all surviving fractions for each experiment separately. Experiments were repeated three times (X-ray) or twice (LD protons). The results across experiments were averaged.

### RNA isolation and sequencing

Total RNA was isolated utilizing the RNeasy mini kit (Qiagen) and DNA was removed using RNase-free DNase (Qiagen). RNA quality was assessed using a BioAnalyzer. RNA sequencing was performed at the UCLA Technology Center for Genomics & Bioinformatics (TCGB). RNA-sequencing reads were mapped to the human genome (GRCh38 reference, including alt contigs, decoy and EBV sequences; downloaded from the 1000 Genomes Project) using STAR v2.5.2b^57^, default parameters. For each replicate, per-gene counts of uniquely mapped reads were computed using HTSeq 0.6.1p2^58^ and Gencode v26^59^ primary assembly annotations. Differential expression analysis was performed and normalized gene counts were generated using DESeq2 v1.16.1^60^. Gene-level enrichment analysis was performed using WebGestalt^61^.

## Supplementary Information

Supplementary Video 1.

Supplementary Video 2.

Supplementary Video 3.

Supplementary Table 1 and Figures.

Supplementary Table 2.

## Data Availability

Data generated or analyzed during this study are included in this published article and its Supplementary Information files. RNA-sequencing data are available from the NCBI SRA under BioProject accession number PRJNA758989.
