# Supplementary material for: A new platform for ultra-high dose rate radiobiological research using the BELLA PW laser proton beamline
Source: Sci Rep. 2022 Jan 27;12:1484. doi: 10.1038/s41598-022-05181-3 (PMC8795353; doi:10.1038/s41598-022-05181-3)
Supplement: Supplementary file 4 — Supplementary Table 1 and Figures. [file 41598_2022_5181_MOESM4_ESM.docx]

**Supplementary materials**

**A new platform for ultra-high dose rate radiobiological research using the BELLA PW laser proton beamline**

Jianhui Bin^1,3^*, Lieselotte Obst-Huebl^1^*, Jian-Hua Mao^2^, Kei Nakamura^1^, Laura D. Geulig^1^, Hang Chang^2^, Qing Ji^1^, Li He^2^, Jared De Chant^1+^, Zachary Kober^1^, Anthony J. Gonsalves^1^, Stepan Bulanov^1^, Susan E. Celniker^2^, Carl B. Schroeder^1^, Cameron G.R. Geddes^1^, Eric Esarey^1^, Blake A. Simmons^2^, Thomas Schenkel^1^, Eleanor A. Blakely^2^, Sven Steinke^1^^ and Antoine M. Snijders^2,#^

^1^Accelerator Technology and Applied Physics Division, Lawrence Berkeley National Laboratory, Berkeley, CA 94720, USA.

^2^Biological Systems and Engineering Division, Lawrence Berkeley National Laboratory, Berkeley, CA 94720, USA.

^3^State Key Laboratory of High Field Laser Physics and CAS Center for Excellence in Ultra-intense Laser Science, Shanghai Institute of Optics and Fine Mechanics, Chinese Academy of Sciences, Shanghai, 201800, China.

* Contributed equally to this work

^ Currently at Marvel Fusion GmbH, Blumenstrasse 28, 80331 München, Germany

+ Currently at Michigan State University, East Lansing, Michigan 48824, USA

**Supplementary Table 1. Proton energy loss simulated with SRIM in each absorber layer along the proton beam path until the cell layer is reached.** Absorber boundaries (start/end), energy at the beginning (energy start) and at the end (energy end) of the layer and time of flight (TOF) of the respective energy classes at the beginning (TOF start) and the end (TOF end) with respect to absolute time 0 at initial acceleration of the protons at the target, and the difference between TOF start and TOF end, representing the pulse length at the end of the absorber, are listed. Given the accuracy of determining the initial energy range applied to the cells (+/- 0.1 MeV), the final pulse length at the location of the cells can be determined as 31 – 35 ns.

| **Layer** |  | **start / mm** | **end / mm** | **energy start / MeV** | **energy end / MeV** | **TOF start / ns** | **TOF end / ns** | **pulse length end / ns** |
| --- | --- | --- | --- | --- | --- | --- | --- | --- |
| Vacuum |  | 0 | 1634 | 0 | 2.9 | 0.0 | 69.3 |  |
| Vacuum |  | 0 | 1634 | 0 | 8 | 0.0 | 41.7 | 27.6 |
| Kapton window |  | 1634 | 1634.025 | 2.9 | 2.5 | 69.3 | 69.3 |  |
| Kapton window |  | 1634 | 1634.025 | 8 | 7.8 | 41.7 | 41.7 | 27.6 |
| Air gap |  | 1634.025 | 1731.025 | 2.5 | 0.5 | 69.3 | 74.8 |  |
| Air gap |  | 1634.025 | 1731.025 | 7.8 | 7.2 | 41.7 | 41.8 | 33.0 |
| Mylar window |  | 1731.025 | 1731.061 | 0.5 | 0.3 | 74.8 | 74.8 |  |
| Mylar window |  | 1731.025 | 1731.061 | 7.2 | 7.2 | 41.8 | 41.8 | 33.0 |

**Supplementary Table 2. Dose and survival measurements for PC3 and RWPE1 after LD proton and X-ray exposure.**

**
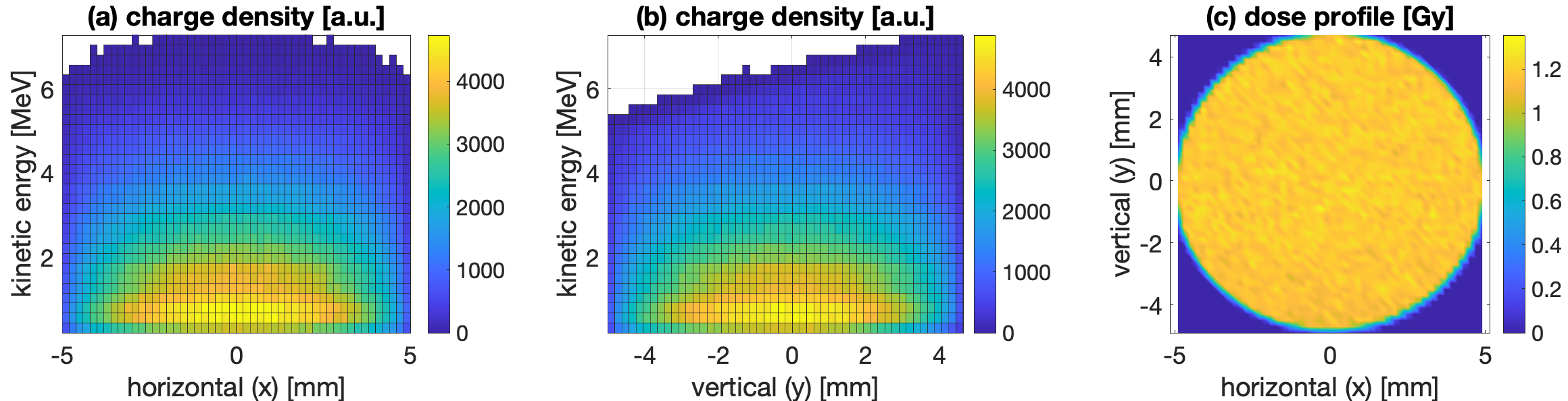
**

**Supplementary Figure 1. Simulated two-dimensional proton spectrum.** (a,b) Simulated two-dimensional (2D) proton spectrum on the cell for horizontal (a) and vertical (b), respectively. (c) Simulated 2D dose profile on the cell, where the standard deviation was 18%. The dose contribution from protons 5.4 MeV and above was 0.6%.

**
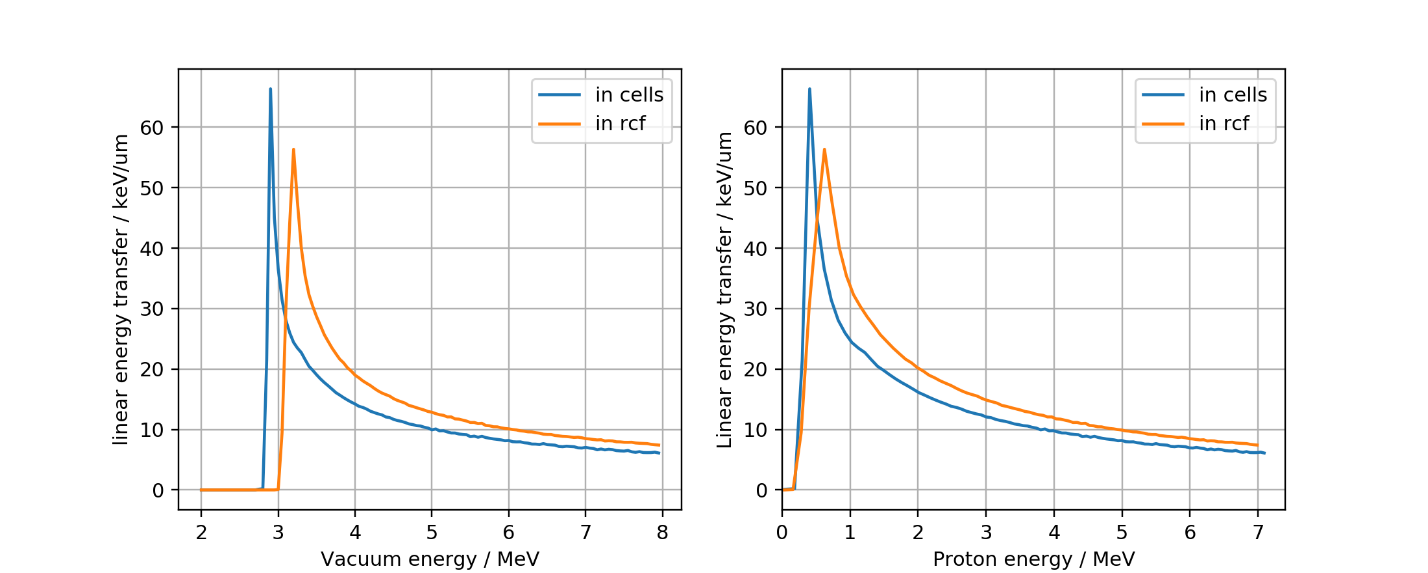
**

**Supplementary Figure 2. Linear energy transfer in cell sample and RCF layer.** Both cells and RCF are exposed to broad energy spectra with varying linear energy transfer across that energy range. X-axis values represent energies after passing through absorbers before reaching the cell sample and RCF layer respectively.

**
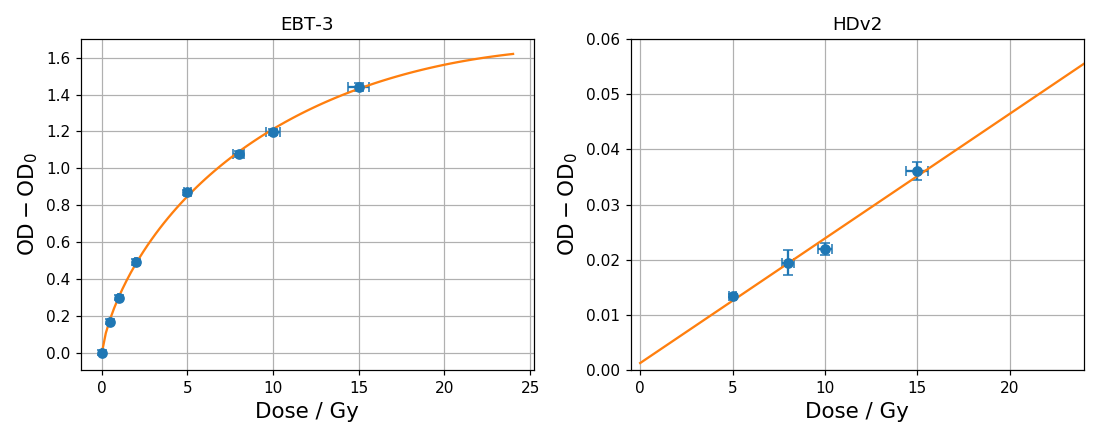
**

**Supplementary Figure 3. Calibration curve of radiochromic films with 300 kVp X-rays for doses between 5 and 15 Gy.** Low dose calibration of EBT-3 (left) and HD-v2 (right) radiochromic films with a 300 kV X-ray tube. OD error bars indicate the standard deviation of the mean optical density (OD) over three samples per dose. Dose error bars represent the calibration accuracy of the reference ionization chamber.


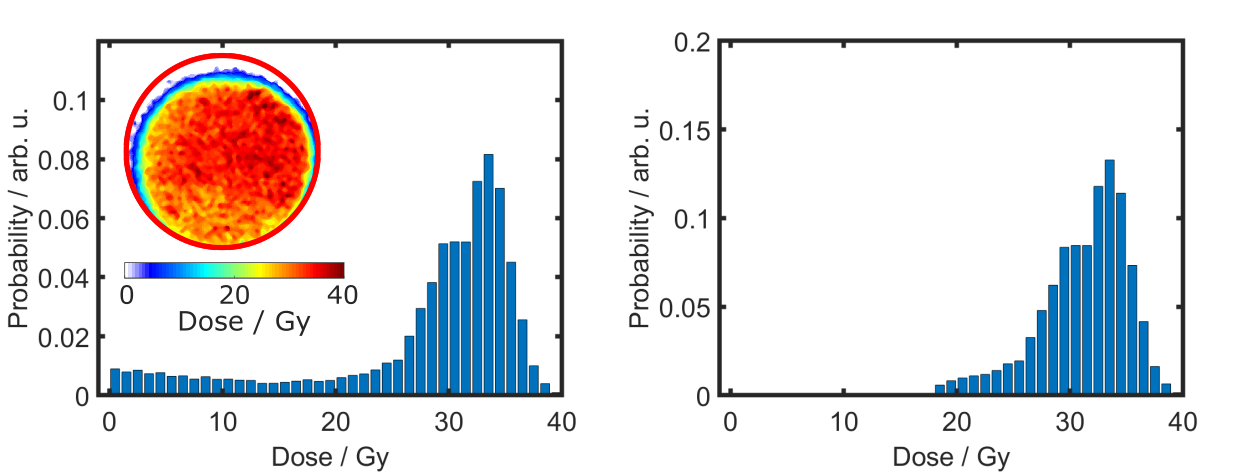


**Supplementary Figure 4. High proton dose for referencing of the background surviving cell fraction due to geometric shadow cast on cell sample.** Average dose of 34 Gy leads to killing of all cells across the cell cup except for those that were located in the shadowed region. Left: dose histogram before subtraction of shadowed region. Right: dose histogram after subtraction of shadow region, the size of which is based on geometry considerations and the fraction of surviving cells.

**Supplementary Video 1. Exploded view of cell culture assembly.**

**Supplementary Video 2. Cell culture assembly mylar membrane application.**

**Supplementary Video 3. Linear motorized stage can hold up to eight cell culture assemblies.**
